# Supplementary material for: Application of Self-Assembly Nanoparticles Based on DVDMS for Fenton-Like Ion Delivery and Enhanced Sonodynamic Therapy
Source: Biosensors (Basel). 2022 Apr 18;12(4):255. doi: 10.3390/bios12040255 (PMC9025210; doi:10.3390/bios12040255)
Supplement: Supplementary file 1 [file biosensors-12-00255-s001.zip › biosensors-1636596-supplementary.pdf]

biosensors

MDPI

Application of Self-Assembly Nanoparticles Based on DVDMS for Fenton-Like Ion Delivery and Enhanced Sonodynamic Therapy

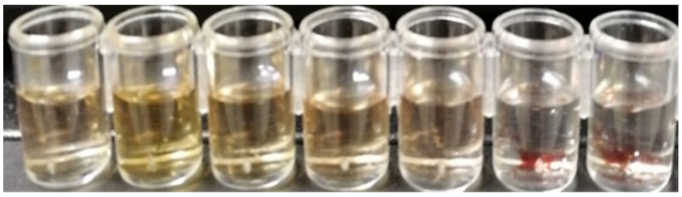

Figure S1. The picture of the DVDMS after reaction with different amounts of Cu^{2+} ($[Cu^{2+}]/[DVDMS]$ ratios (0: 1, 0.5: 1, 1: 1, 2: 1, 5: 1, 10: 1 and 20: 1).

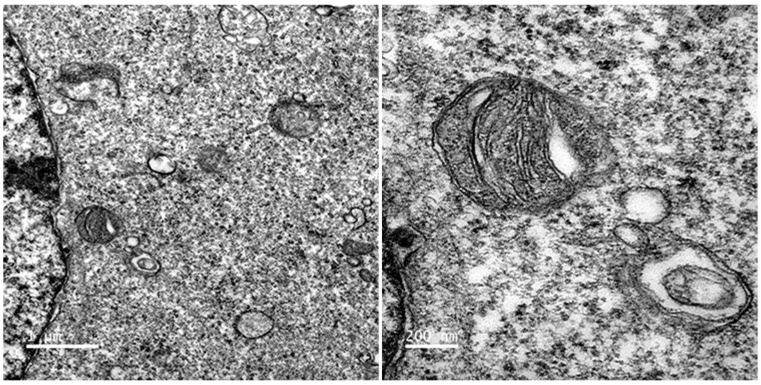

Figure S2. Cell TEM image of the cells incubated with DVDMS at 12 h.

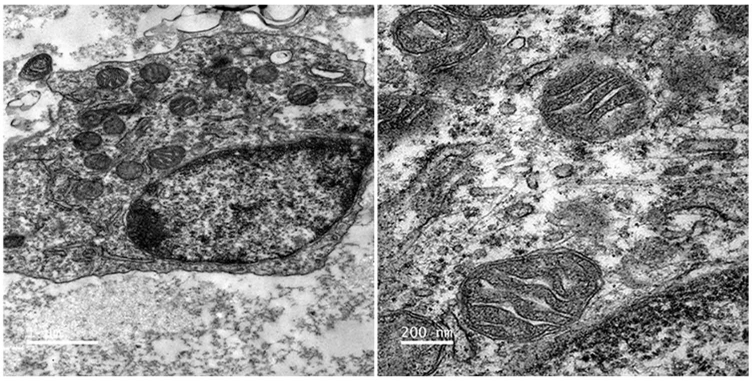

Figure S3. TEM images of 4T1 tumor thin sections at 12 h after injection of DVDMS.

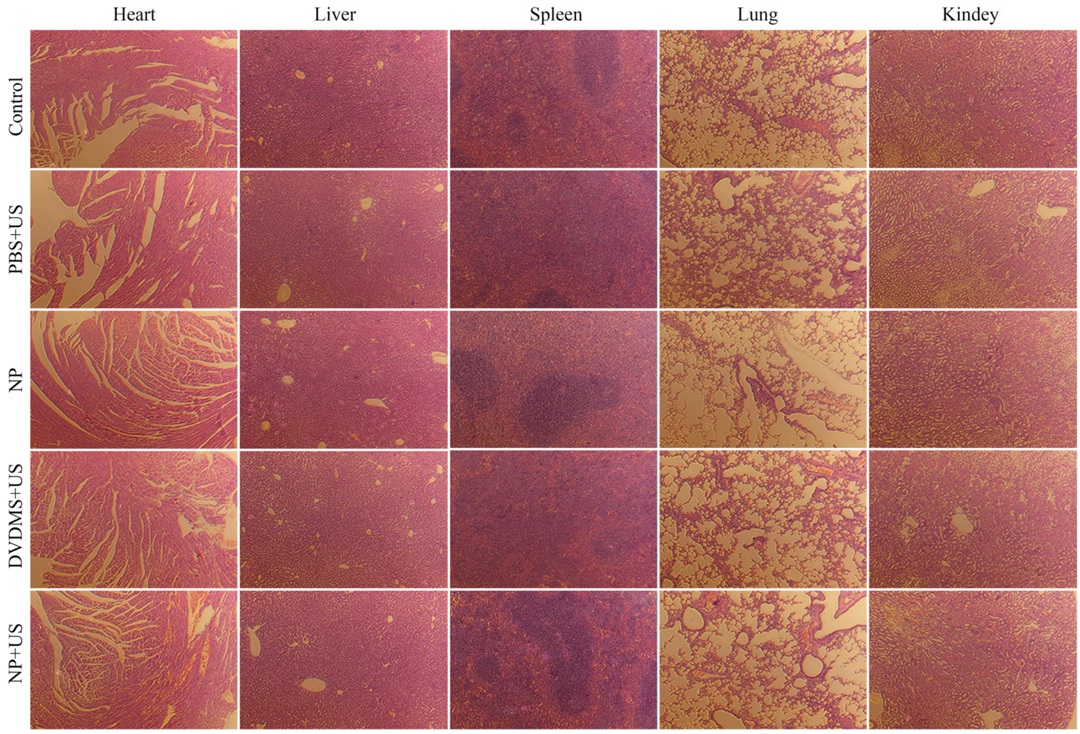

Figure S4. Histochemical study of the organs harvested from mice with different treatments at 12 h.

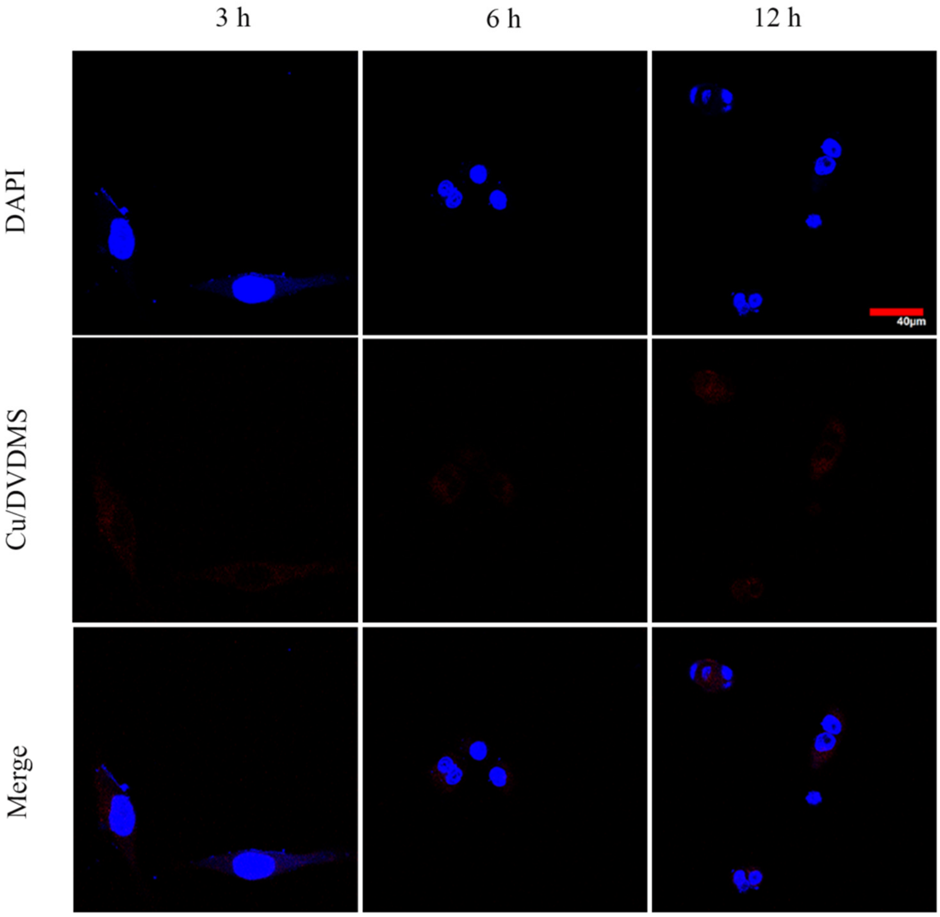

Figure S5. The CLSM images of 4T1 cells incubated with Cu/DVDMS (DVDMS concentration was 10 μg/mL) at 3, 6, 12 h. Scale bar = 40 μm.

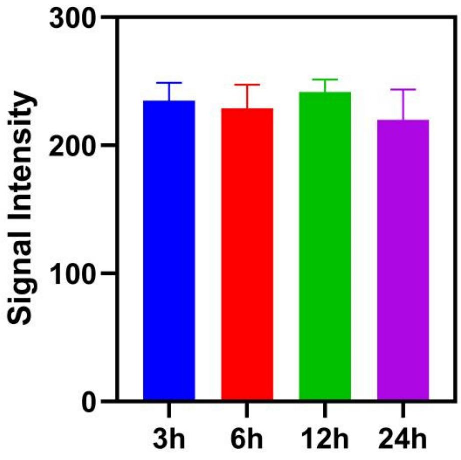

Figure S6. Quantitative analysis of PET signal in tumor section at 3, 6, 12, 24 h.
